# Analysis of the 5'UTR of HCV genotype 3 grown *in vitro *in human B cells, T cells, and macrophages

**DOI:** 10.1186/1743-422X-7-155

**Published:** 2010-07-13

**Authors:** Dennis Revie, Michael O Alberti, John G Prichard, Ann S Kelley, S Zaki Salahuddin

**Affiliations:** 1 Department of Biology, California Lutheran University, Thousand Oaks, California, USA; 2 Ventura County Medical Center, Ventura, California, USA; 3 Ventura County Hematology-Oncology Specialists, Oxnard, CA, USA; 4 California Institute of Molecular Medicine, Ventura, California, USA; 5 Division of Human Gene Therapy, Department of Medicine, University of Alabama at Birmingham, Birmingham, AL, USA

## Abstract

**Background:**

Previously, we have reported the isolation and molecular characterization of human Hepatitis C virus genotype 1 (HCV-1) from infected patients. We are now reporting an analysis of HCV obtained from patients infected with HCV genotype 3 (HCV-3) as diagnosed by clinical laboratories.

**Results:**

HCV was cultured *in vitro *using our system. HCV RNA was isolated from patients' blood and from HCV cultured in various cell types for up to three months. The 5'UTR of these isolates were used for comparisons. Results revealed a number of sequence changes as compared to the serum RNA. The HCV RNA produced efficiently by infected macrophages, B-cells, and T-cells had sequences similar to HCV-1, which suggests that selection of the variants was performed at the level of macrophages. Virus with sequences similar to HCV-1 replicated better in macrophages than HCV having a 5'UTR similar to HCV-3.

**Conclusions:**

Although HCV-3 replicates in cell types such as B-cells, T-cells, and macrophages, it may require a different primary cell type for the same purpose. Therefore, in our opinion, HCV-3 does not replicate efficiently in macrophages, and patients infected with HCV-3 may contain a population of HCV-1 in their blood.

## Background

HCV is associated with a number of diseases, including hepatocellular carcinoma, B-cell lymphomas, and neuropathy. There is an emerging list of diseases that may have some association with this virus. Approximately 8% of HCV-infected individuals in the United States are infected with genotype 3 [1]. The chances of liver damage due to HCV infections may not vary by genotype in untreated individuals [2,3], and infections with HCV-3 are more likely to respond earlier to ribavirin/α-interferon combination therapy than HCV-1 [3-5]. There is evidence that individuals infected with HCV-3 are likely to progress rapidly to liver steatosis [6], and fibrosis [7] compared to infection with HCV-1. Individuals infected with HCV are also frequently infected with other viruses. Hematopoietic cells e.g., HCV infected T-cells, are capable of being co-infected with HIV-1 and HHV-6 [8]. All of the co-infecting viruses continue to replicate in these cells.

Although synthetic constructs are commonly used for HCV related studies, we have a system for studying the natural virus isolated from infected patients. Reports using constructs viz., Replicon, pseudo-particles etc. may have produced interesting data, but these results lack meaning in the area of human diseases and public health. Meaningful data must come from viruses isolated from patients since no Replicon disease yet exists.

The 5'UTR of HCV controls replication through cap-independent translation [9-11]. For this report, HCV was isolated from the blood of patients infected with HCV-3 and transmitted into macrophages, B-cells, and T-cells. The 5'UTR of the progeny viruses was analyzed and compared to the sequences of the HCV RNA found in these patients' sera. In our previous reports, the 5'UTR of HCV-1 isolates from patients was analyzed and compared to HCV cultured *in vitro *and minor differences were found between the HCV in the isolates and patients' sera [12,13]. This suggested that the virus in culture was similar to that found in patients' blood.

## Results

**Figure 1:**
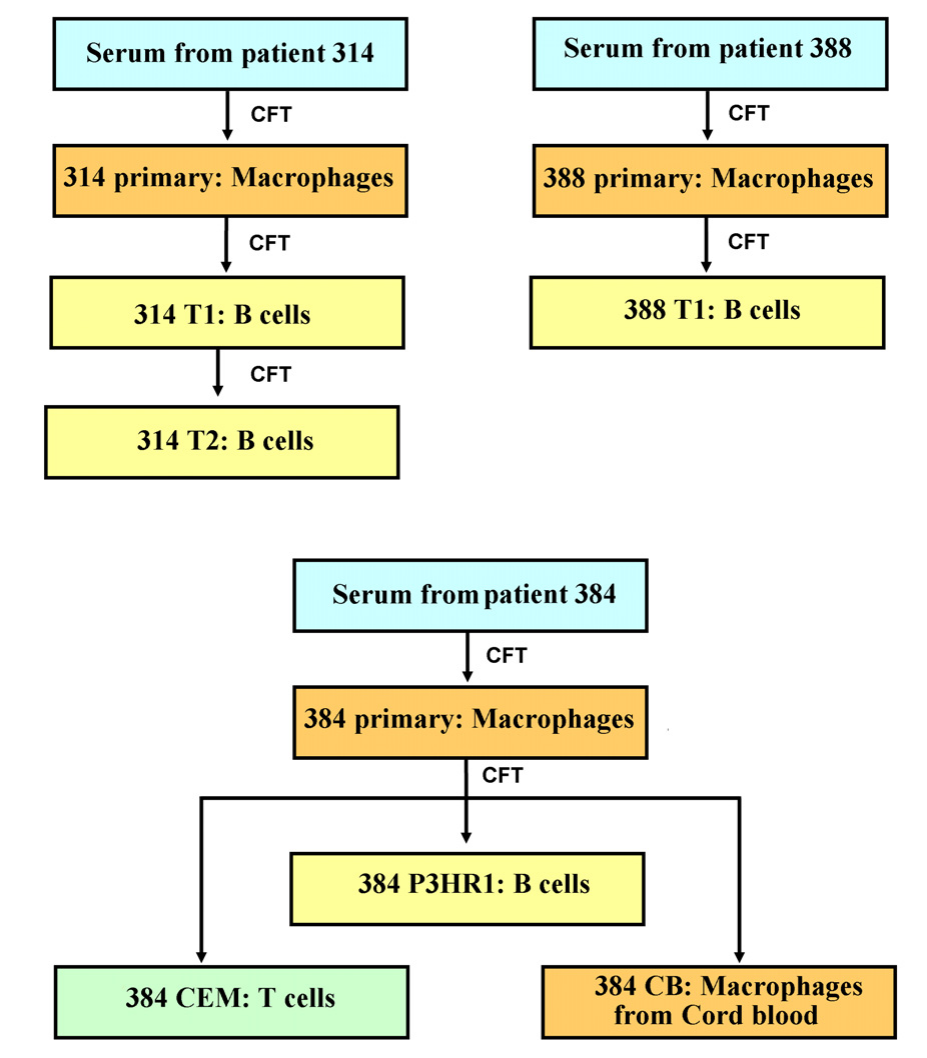
**Flow chart of HCV isolation at CIMM**. Samples in the boxes were sequenced and analyzed for this report. Cell-free transfers (CFT) of HCV into freshly prepared cells are indicated by arrows. Cell types are indicated by colors.

The data reported here represents the analysis of the 5'UTR of HCV-3 from three patients (designated samples 314, 384, 388). Primary and secondary isolates analyzed for the study were cultured *in vitro *for up to three months and compared to the HCV RNA from the patients' sera. The primary isolates are obtained from cultured macrophages. A flow chart of our samples is shown in Figure 1.

### Determination of best primer sets for analyses

**Figure 2:**
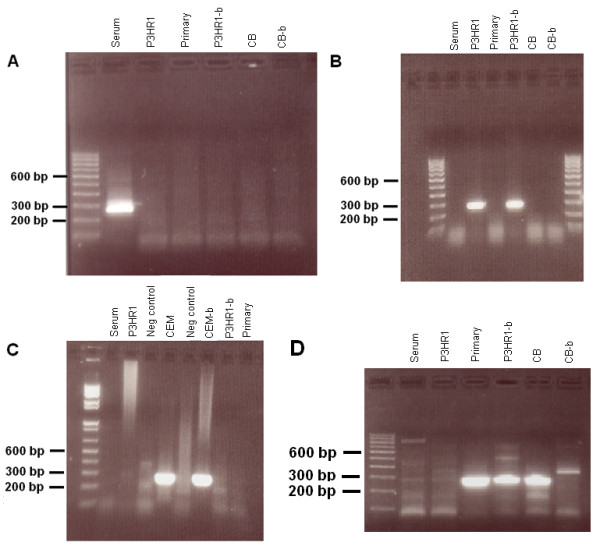
**Gel electrophoresis of patient 384 RT-PCR products using various sets of primers**. For short time cultures and analyses, we have used cloned cell lines such as P3HR1 and CEM. This was for the purposes of reproducibility and economy. A. Primer sets 9.1a-9.2a (1^st ^PCR) and 37up-318down (2^nd ^PCR). Only 384 serum produced a positive band. CB designates Macrophages purified from cord blood. P3HR1-b and CB-b were supernatants collected at different times than P3HR1 and CB. B. Primer sets 9.1a-9.2a (1^st ^PCR) and 10.1a-10.2 (2^nd ^PCR). Only P3HR1 produced positive bands. C. Primer sets 8up-347down (1^st ^PCR) and 37up-318down (2^nd ^PCR). First PCR at 50°C. Only CEM produced positive bands. D. Primer sets 8up-347down (1^st ^PCR) and 37up-318down (2^nd ^PCR). First PCR at 55°C. Primary, P3HR1-b, and Cord blood macrophages produced positive bands.

For the purposes of this report, we first made degenerate versions of the primers that were used for HCV-1 [12]. These primers were named 9.1a, 9.2a, and 10.1a (Table 1). Although these sets of primers were suitable for some HCV-3 samples (Figure 2B), we were unable to obtain the appropriate reactivity for the remainder of the samples. We therefore designed another set of primers to work with genotypes 1 and 3 (named 8 up and 347 down for the first PCR; 37 up and 318 down for the second PCR). Unlike our HCV-1 samples, we were unable to find a single set of primers and PCR conditions that always worked with all of the HCV-3 samples. By testing different combinations of primers and temperature conditions, we were able to generate PCR fragments for all of our samples (Figure 2 and Table 2).

**Table 1:** List of primers for this study.

Primer	Strand	Starting position	Ending position	Sequence (5' to 3')
9.1a	Positive	24	42	GAC ACT CCA CCA TRG ATC ACT C

9.2a	Negative	344	323	CAT GWT GCA CGS TCT ACG AGA C

10.1a	Positive	48	71	CTG TGA GGA ACT WCT GTC TTC ACG CRG

10.2	Negative	310	293	CAC TCG CAA GCA CCC TAT CAG

9.1a-flap	Positive	24	42	AAT AAA TCA TAA GAC ACT CCA CCA TRG ATC ACT C

9.2a-flap	Negative	344	323	AAT AAA TCA TAA CAT GWT GCA CGS TCT ACG AGA C

8 up	Positive	11	28	CCC TGA TGG GGG CGA CAC TCC

347 down	Negative	345	325	TGC TCA TGG TGC ACG GTC TAC GAG

37 up	Positive	41	66	CAC TCC CCT GTG AGG AAC TAC TGT CTT CA

318 down	Negative	316	295	CCG GGG CAC TCG CAA GCA CCC TAT C

The starting and ending positions are relative to HCV H77 [26].

**Table 2:** List of samples and PCR conditions in this study.

Isolate	Cell type	Date of transmission	Clones sequenced	Outer PCR primers	**Outer PCR Temp**.	Inner primers	**Inner Temp**.
314 serum	All	1/5/05	25	9.1a-9.2a	55	10.1a-10.2	60

314 T1	B cell	1/13/05	17	9.1a-9.2a	55	10.1a-10.2	60

314 T2	B cell	2/4/05	5	9.1a-9.2a	55	10.1a-10.2	60

384 serum	All	1/8/08	24	8 up-347 down	50	37 up-318 down	55

384 primary	Macrophage	1/30/08	26	8 up-347 down	55	37 up-318 down	55

384 P3HR1	B cell	1/30/08	25	9.1a-flap-9.2a-flap	50	10.1a-10.2	60

384 CEM	T cell	1/30/08	27	8 up-347 down	50	37 up-318 down	55

384 CB	Macrophage	4/7/08	8	8 up-347 down	55	37 up-318 down	55

388 serum	All	9/10/08	6	8 up-347 down	50	37 up-318 down	60

388 primary	Macrophage	9/12/08	5	8 up-347 down	50	37 up-318 down	60

388 T1	B cell	9/18/08	4	8 up-347 down	50	37 up-318 down	60

### Comparison of the 5'UTR of HCV from patients' sera and CIMM-HCV

**Figure 3:**
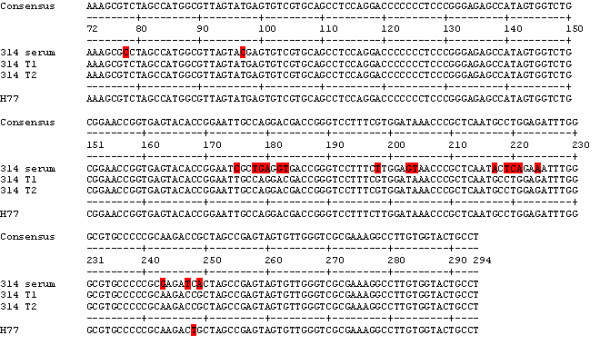
**Comparisons of 5'UTR consensus sequences between patient 314 serum and isolates of HCV**. H77, GenBank accession number NC_004102, is shown for comparison purposes and was not used to determine the consensus.

The first sample of HCV-3 analyzed was from patient 314. Few or no changes in the sequences of the 5'UTR were observed for cultured HCV-1, but 19 changes in the sequences for patient 314 RNA were observed (Figure 3). The sequences for 314 T1 and 314 T2 were the same, showing that consecutive transfers of HCV into the same cell type do not affect the sequence. The 314 T1 and T2 sequences were almost identical to genotype 1 H77, therefore the isolation system for HCV-3 replicated virus which had a 5'UTR similar to HCV-1. This was unexpected, but suggested that there were some HCV-1 in the blood of patient 314 that preferentially replicated in macrophages.

**Figure 4:**
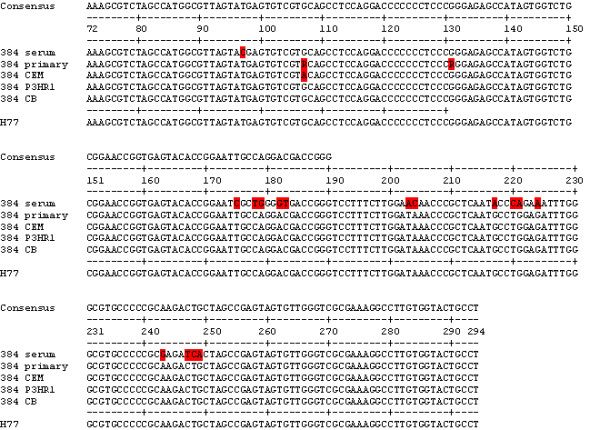
**Comparisons of 5'UTR consensus sequences between patient 384 serum and isolates of HCV**. H77 is shown for comparison purposes and was not used to determine the consensus.

Due to the data from patient 314, HCV was isolated and sequenced from patient 384. When comparing 384 serum RNA with the four HCV isolates from that serum, there were approximately 17 changes for each isolate (Figure 4), with many of these changes identical to those found in the isolates from patient 314. Furthermore, since there were only small differences between primary isolates (cultured in macrophages) and the secondary isolates (cultured in B-cells and T-cells), the macrophages appear to have selected HCV with a particular sequence that most closely matched HCV-1. It is possible that patient 384 also had low levels of HCV-1, which was amplified in culture. Although the sequences in the 5'UTR that we are reporting for the isolates were almost identical to H77, we could not amplify the region using our standard primers for HCV-1 samples (9.1a and 9.2a then 10.1a and 10.2, Table 1). It may be that the region upstream or downstream of the reported sequences is similar to HCV-3.

**Figure 5:**
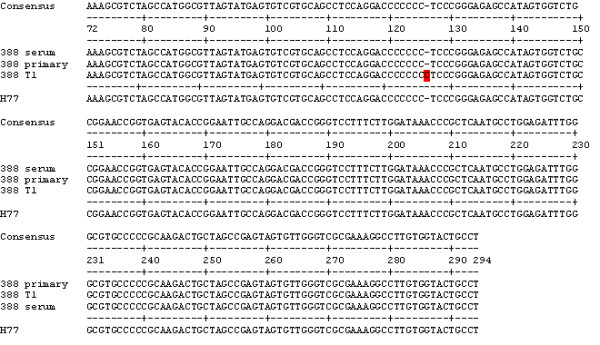
**Comparisons of 5'UTR consensus sequences between patient 388 serum and isolates of HCV**. H77 is shown for comparison purposes and was not used to determine the consensus.

**Figure 6:**
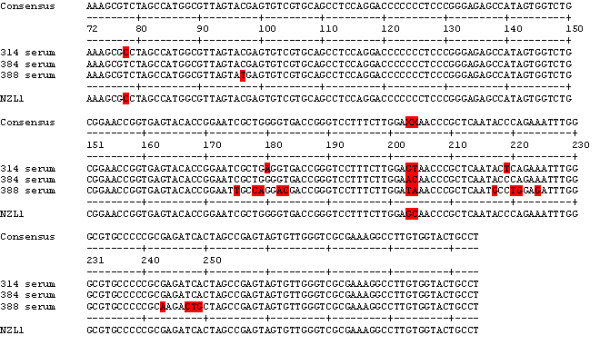
**Comparisons of 5'UTR consensus sequences of sera from patients 314, 384, and 388**. NZL1 is a reference HCV-3 at NCBI Entrez genome (accession number NC_009824), is shown for comparison purposes and was not used to determine the consensus sequence.

Next, we isolated and sequenced samples from another patient infected with HCV genotype 3: patient 388. Comparing the serum and the isolates did not reveal large changes in the sequence. In fact, the only change observed was an additional C in a string of C's from bases 121 to 126 for sample 388 T1 (Figure 5). A comparison of sera from patients 314, 384, and 388 revealed that the 388 5'UTR sequence is actually different from the other two patient serum sequences (Figure 6). Comparisons of the 388 5'UTR sequence and other HCV sequences show that it is similar to the 5'UTR of HCV-1. Therefore, although a clinical lab typed the patient samples as being infected with HCV-3 based on a standard methodology, our results suggest the presence of HCV-1, as well. This may have been a clinical testing error. The sequence found in the macrophages was the same as in the serum.

Other investigators have found that HCV-3 5'UTR sequences direct translation in an *in vitro *system about as efficiently as HCV-1 sequences [14]. However, a recent report by others compared HCV-1 and HCV-3 5'UTR via Replicons, and showed that HCV-3 versions replicate more slowly in their system than HCV-1 [15]. This agrees with our results that HCV with genotype 1 sequences preferentially replicate in our system.

### Analysis of the sequence variability

Our previous analyses of the sequence variability of the 5'UTR of HCV-1 found a small increase in the variability of cultured HCV compared to patients' HCV RNA [13]. To see if this also applies to cultured HCV-3, we determined the variability of the 314 and 384 HCV-3 samples (Table 3).

**Table 3:** Sequence complexity of genotype 3 HCV samples.

Sample	Number	Shannon entropy (Sn)	Pn (10-3)
314 serum	26	0.50	2.46

314 T1	17	0.15	0.11

384 serum	24	0.26	2.01

384 primary	26	0.53	0.84

384 P3HR1	25	0.26	1.05

384 CEM	27	0.41	1.14

Pn variability and Shannon entropy, normalized for the number of samples, were calculated as described by Cabot *et al. *[27] and Pawlotsky *et al. *[28].

**Figure 7:**
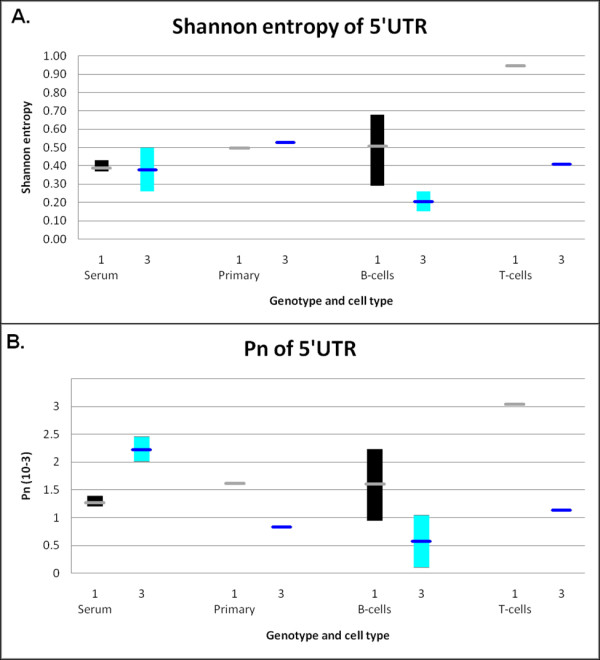
**Sequence complexity of genotype 3 HCV samples**. (A) Shannon entropy comparisons of genotypes 1 and 3 HCV cultured in various cell types. (B) The Pn variability of genotype 1 and 3 HCV cultured in various cell types. The genotype 1 values are from Revie et al. [13]. The ranges are indicated by vertical bars and the means by thick horizontal lines.

Shannon entropy is a measure of the number of different genomes in the sample. Genotype 1 and 3 serum and primary samples have approximately the same Shannon entropy (Figure 7A). However, HCV-3 cultured in B-cells and T-cells had lower Shannon entropy than HCV-1, suggesting that particular genomes replicate better in these cells compared to macrophages. We are in the process of determining if longer culturing affects the variability.

Pn is a measure of the number of polymorphic sites in the genome, and is proportional to the number of nucleotide positions that vary in the genome. The genotype 3 serum samples have a greater Pn than genotype 1 samples (Figure 7B). However, the HCV-3 primary, B-cell, and T-cell HCV samples have lower Pn than HCV-1 samples. This limited analysis agrees with the Shannon entropy data in that the variability of the cultured HCV-3 is lower than HCV-1.

### Distribution of variant bases in isolated HCV consensus sequences

**Figure 8:**
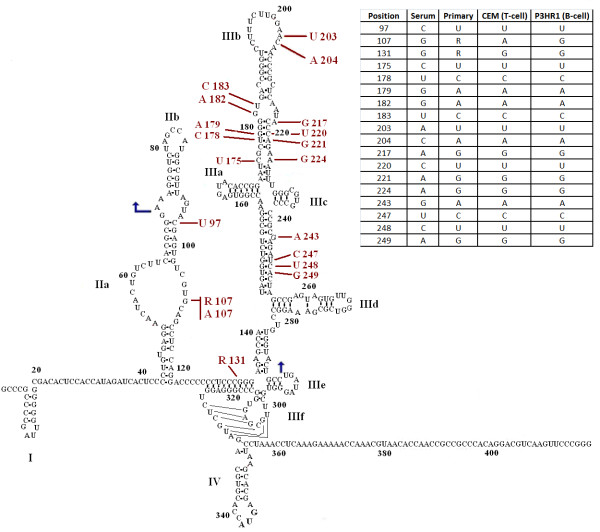
**Variation in the bases of 384 isolates**. The 384 serum sample is used for the figure, with sequence changes found in the isolates indicated. The table lists each isolate and the base at each position in the sequence. The arrows indicate the limits of the reported sequences. The figure is adapted from Lyons et al. [29].

We compared HCV RNA from patient 384 serum and four laboratory isolates to determine if changes were consistent with the current 2 D model of the 5'UTR RNA proposed by Honda *et al. *(2001). The 19 variant bases were either in regions that are not base paired, or where the changes would not affect base pairing (Figure 8). So although there were a number of changes to the sequence after culturing, these did not appear to cause any differences that would affect the overall 2 D structure of this model. All of the differences in the stems had either compensatory changes (e.g. GC to AU at bases 179 and 220) or could use alternative base pairing (e.g., GC to GU at bases 145 and 248). We are not sure how these changes impact the replication of HCV.

## Discussion

There are numerous reports about differences between different strains or types of HCV. We are reporting the isolation and replication of HCV from patients infected by type 3 strains of HCV. These new isolates can be cultured in both B and T cells. By contrast to type 1 strains of HCV, sequence comparisons of the 5'UTR of HCV found in patients' sera and their corresponding *in vitro *isolates suggests significant changes in the sequences of type 3 strains. The replication of other HCV genotypes such as 2, 4, 5, and 6 in our system is awaiting studies.

As we have published before, macrophages are important in selecting HCV-1 for replication [12,16]. Since these cells are focus of our studies, we would like to name them as cells that are functionally highly phagocytic and cytochemically stain intensely for non-specific esterase. This would include both fixed and free cells such as histiocytes in connective tissue, Kupffer cells of liver, microglial cells of neuronal tissue, dendritic cells of skin, and alveolar macrophages to name a few. The presence of HCV in monocytes or macrophages has been shown in HCV-infected individuals [17-21]. In addition, like HIV, infected macrophages may act as a reservoir of biologically active, infectious HCV *in vivo*. Although, we have had some success in isolating HCV-3, the system is not optimal. Other types of macrophages, e.g. Kupffer cells, are probably better for replication of genotype 3. Results presented here show that we were able to isolate and culture HCV from patients infected with HCV-3 to a limited extent. However, the HCV-3 produced by macrophages, B-cells, and T-cells were significantly different from HCV-3 in the patients' sera (Figures 3 and 4).

We were unable to find one set of PCR primers and conditions that worked for all of our samples. For genotype 1, we routinely use the same set of conditions for the analysis of the 5'UTR. For genotype 3, we found that some samples would not work for any particular set of PCR conditions. This is presumably due to a high degree of variability of HCV-3.

Our studies indicate that the macrophages preferentially select HCV-1, making them the dominant virus type (Figure 4), and HCV-3 may poorly infect macrophages from cord blood. The reduced sequence complexity of HCV-3 cultured in B-cells and T-cells suggests that macrophages are selecting against this genotype. Sera from patients 314 and 384 had HCV-3 sequences, while the HCV in macrophages and other cell types was only HCV-1.

The 388 T1 sample (B-cells) had 8 C's starting at position 120, compared to 7 for the 388 serum and 388 primary (macrophage) samples. Although we only sequenced four clones for this sample, every one of them had an extra C. We have previously observed an additional C in several HCV-1 samples [13]. In addition, we have found a large deletion in this area for one sample [16]. This region is located between stem-loops II and III, thus apparently allowing greater variability. HCV needs to be infectious, and the level of replication of these infectious agents will depend upon a number of factors, most importantly the target cells.

The results presented here suggest that HCV-3 may need a different cell type for its primary replication *in vitro*. Our previous publications document the selection of HCV-1 in macrophages or similar cells viz., neuronal precursors. Individuals infected with genotype 3 may have small amounts of other genotypes circulating in their blood. It is possible that these other genotypes may also prefer to infect specific cell types for replication *in vitro*. Others have shown that different tissues in one particular individual may harbor different genotypes of HCV [22-24], suggesting that cell tropism may establish the tissue specificity of HCV in infected individuals. Variability in diseases of HCV-infected individuals, such as neuropathy and lymphoma, may either be due to variations in the virus or to increased susceptibility of infected cell types, or the presence of other viral agents in circulation. This phenomenon is under further investigation.

## Methods

### Patient samples

All patient samples were given a code at the source, and a sequential number in our laboratory to preserve their anonymity. Patients 314, 384, and 388 were all AIDS patients doubly infected with HIV and HCV. The HCV was genotyped as type 3 using an INNO-LiPA assay by a clinical testing laboratory (Quest Diagnostics).

### *In vitro *culture system

Our culture system, described earlier, takes advantage of the infectious particles present in the peripheral circulation [12]. Briefly, the isolation of HCV was done in two stages: (A) HCV derived from patients' blood was used to infect human macrophages; (B) HCV obtained from the macrophages was then used to infect freshly transformed B-cells or T-cells obtained from human fetal cord blood and cultured in the presence of 100 units/ml of IL-2 (Collaborative Biomedical Products, catalog number 40121). The types of samples that were analyzed included: (i) HCV found in serum or plasma of patients; (ii) HCV produced by macrophages (primary isolate); and (iii) HCV produced by B-cells or T-cells (secondary isolates). Each sample was given a unique number that indicated the patient and a suffix designating replication into various cell types. Transfers into fresh uninfected B-cells were given a suffix of T1, transfers into cell lines, such as P3HR1 and CEM, were given a suffix of the cell line name, and secondary transfers into macrophages produced from human cord blood (CB). All isolates were produced in the laboratories at CIMM, and are therefore called CIMM-HCV. The data in this paper is based on these isolates.

CEM.NCI and CEM.SS cells were obtained through the NIH AIDS Research and Reference Reagent Program, Division of AIDS, NIAID, NIH. P3HR1 cells were obtained from ATCC. The cell types were cultured using the methods recommended by ATCC. Single cell clones were established from these cell lines for the sake of uniformity of data and the removal of adventitious material such as mycoplasma.

### RT-PCR

RNA was purified using TriReagent as previously described [12], and a nested RT-PCR was performed. For the patient 314 samples, the procedure was as previously described using Fidelitaq (US Biochemicals) [12]. For the other patient samples, RT was performed using N_12 _random primers for 12 12-minute cycles at 48°C using cyclic RT (Bioneer). The PCR was performed using Bioneer high fidelity TLA PCR premixes. The primers used for the experiments are listed in Table 1.

For each TLA PCR reaction, samples were denatured at 94°C for 3 minutes, and then 30 cycles of amplification were performed with the following temperature profiles: 94°C for 30 seconds, 50 or 55°C for 30 seconds, and 72°C for 1 min for the outer primer set and 94°C for 30 seconds, 55 or 60°C for 30 seconds, and 72°C for 1 min for the inner primer set (Table 2). Normally, 2 ml of RNA was used for the RT, 2 ml of the cDNA used for the first PCR, and 2 ml of the first PCR product for the second PCR. Volumes were adjusted as needed.

### Sequencing

Fragments comprising approximately 263 or 276 bp of the 5'UTR generated by nested PCR were cloned using Invitrogen's ZeroBlunt cloning kit. Plasmid DNA from a minimum of 4 clones of each sample were amplified by Templiphi (Amersham) and then sequenced using a Beckman CEQ8000 Genetic Analysis system. In order to ensure high quality analyses, only clones that had identical sequences for both strands were analyzed. All methods followed the manufacturers' protocols.

### Bioinformatics

Analysis of the sequences was performed as described previously [13]. The numbers for the base positions that are reported here are the bases compared to the positions of the full length genome of HCV H77 [25,26].

Complexity of the variation was calculated as Shannon entropy and Pn complexity as described previously [13]. Only samples with at least 17 sequences were analyzed for variation.

### Accession numbers of HCV sequences used for genotyping

The 5' UTR sequences reported in the paper have the GenBank accession numbers HM641722 to HM641732.

## Competing interests

All intellectual rights are reserved by the California Institute of Molecular Medicine (CIMM). There are no competing interests between California Lutheran University or any other body and CIMM.

## Authors' contributions

SZS performed the biological work. JGP and ASK performed the clinical work, recruitment of patients, and procurement of specimens. MOA designed experiments and performed molecular work. SZS and DR designed and conducted experiments, analyzed the data, and wrote the manuscript. All of the authors have read and approved the final manuscript.
